# Modelling tree shape and structure in viral phylodynamics

**DOI:** 10.1098/rstb.2012.0208

**Published:** 2013-03-19

**Authors:** Simon D. W. Frost, Erik M. Volz

**Affiliations:** 1 Department of Veterinary Medicine, University of Cambridge, Madingley Road, Cambridge, Cambridgeshire CB3 0ES, UK; 2 Department of Epidemiology, University of Michigan, Ann Arbor, 1415 Washington Heights, Ann Arbor, MI 48109-2029, USA

**Keywords:** phylodynamics, viral evolution, coalescent, epidemiological models, tree shape

## Abstract

Epidemiological models have highlighted the importance of population structure in the transmission dynamics of infectious diseases. Using HIV-1 as an example of a model evolutionary system, we consider how population structure affects the shape and the structure of a viral phylogeny in the absence of strong selection at the population level. For structured populations, the number of lineages as a function of time is insufficient to describe the shape of the phylogeny. We develop deterministic approximations for the dynamics of tips of the phylogeny over evolutionary time, the number of ‘cherries’, tips that share a direct common ancestor, and Sackin's index, a commonly used measure of phylogenetic imbalance or asymmetry. We employ cherries both as a measure of asymmetry of the tree as well as a measure of the association between sequences from different groups. We consider heterogeneity in infectiousness associated with different stages of HIV infection, and in contact rates between groups of individuals. In the absence of selection, we find that population structure may have relatively little impact on the overall asymmetry of a tree, especially when only a small fraction of infected individuals is sampled, but may have marked effects on how sequences from different subpopulations cluster and co-cluster.

## 1. Introduction

Viruses, especially RNA viruses such as human immunodeficiency virus type 1 (HIV-1), hepatitis C virus and influenza A virus, may exhibit a great deal of genetic variation at the population level, allowing the reconstruction of viral phylogenies that reflect the past transmission of the virus. The shape of the phylogeny can tell us a great deal about how population processes, such as changes in population size and geographical population structure, and immunological processes, such as selection on the virus to escape immune responses, interact [1]. For example, ‘star-like’ phylogenies are typical of populations that are growing exponentially, while ‘ladder-like’ phylogenies are consistent with a model where one variant is replaced by another due to immune escape. This integration of ecological, epidemiological and evolutionary processes has been dubbed ‘phylodynamics’ [2]. Phylodynamic approaches have been used in hundreds of studies of viruses [3] and have generated important insights into the transmission dynamics of many viral pathogens, such as the spread of HIV in the UK [4,5], as well as the geographical spread of influenza A [6–8]. The information obtained by applying phylodynamic models to viral sequence data would be hard, if not impossible, to obtain through more classical epidemiological approaches.

The majority of phylodynamic studies have employed models derived from simple population dynamic models of single species. However, these models may be inappropriate when considering the spread of a virus in a population. A key quantity in these models is the *coalescence rate*, the rate at which lineages coalesce in a phylogeny as we go backwards in time from the present. From the coalescence rate, these models generate estimates of *effective population size* or *N*_e_, which is commonly (mis)interpreted as being proportional to the number of infected individuals. Previously, we have demonstrated that the coalescence rate of an infectious disease is related not only to the prevalence, but also to the rate of transmission (i.e. the incidence) [9]. Consequently, the conclusions of previous studies, particularly those that integrate viral sequence data with information on prevalence, may have to be reinterpreted. The use of epidemiological models to underpin viral evolutionary models can lead to results that are more easily interpretable, and permit the inclusion of prior information, such as that on the duration of the infectious period, as well as facilitating the integration of phylogenetic data with other forms of epidemiological data [10].

Recently, there has been increased interest in considering the phylodynamics of structured populations, for example, in the context of studying the spatial spread of viruses from sequence data (‘phylogeography’) [11]. In addition, there are many other forms of heterogeneity that may be important, including differences by age, duration of infection, contact rate, infectiousness, susceptibility, treatment or vaccination status, etc., depending on the system being studied. When data are available on which subpopulation a viral sequence is associated with, there are a variety of tests that can be used to assess whether there is significant population structure (see Zárate *et al.* [12] for a comparison of several approaches to within-host HIV population structure). A particular challenge arises when data on the subpopulations are lacking. For example, while acute HIV infection is associated with higher infectiousness, information on the time since infection may not be available; similarly, while there may be differences in contact rates between different subpopulations, many molecular epidemiological studies of HIV do not collect behavioural data. Recently, Leventhal *et al.* [13] took an inventive approach to this problem; using a phylogeny from the Swiss HIV epidemic, they showed that the phylogeny was significantly more unbalanced than expected from a simple model of random mixing, which they argued could be due to contact structure in the at-risk population. They used a measure of tree balance, Sackin's index [14], and derived an approximation of the expectation of Sackin's index given a transmission network. Of note, they did not present a similar approximation for Sackin's index given the underlying *contact network*.

In this study, we consider how population structure may affect phylodynamic patterns, using HIV-1 as a model system. We first introduce the notion of tree imbalance or asymmetry. We then review our framework for modelling coalescence using ordinary differential equations, presenting another perspective on our past results, which we extend to consider the dynamics of external branches (or tips or leaves) of the phylogenetic tree. This device allows us to model cherries [15], pairs of tips that share a direct common ancestor, which we use to capture both tree asymmetry as well as population structure. We also derive an approximation to Sackin's index as a complementary measure of tree asymmetry. We apply this approach to determine how (i) higher infectiousness during acute infection and (ii) the presence of a high-risk group with a high contact rate may affect the shape and the structure of the viral phylogeny.

## 2. Asymmetry of phylogenetic trees

The most widely used model used in studies of viral phylodynamics is the time-varying coalescent model [16], which considers the genealogical process in a population that changes size in a deterministic fashion according to some relative size function, *ν*(*τ*), where *τ* is time measured in generations, starting with the present and going backwards. Assuming a sample of *n* individuals taken at time *τ* = 0, and that the sample can be traced back to a single common ancestor with probability one, the dynamics of the number of distinct ancestors of the sample at time *τ* is modelled as a stochastic process 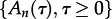, which starts at *A_n_*(0) = *n*, and moves down in steps of 1 until reaching 1, at which point the sample has been traced back to the common ancestor. In a small time-step *h*, the transition probabilities are determined by the following:2.1
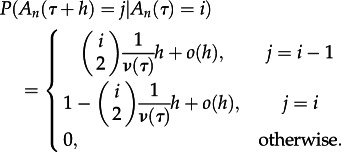


**Figure 1.**
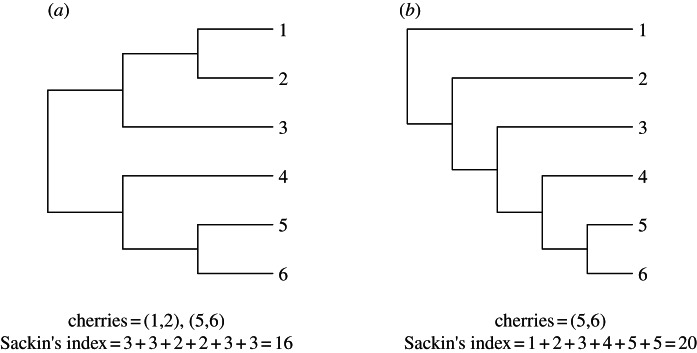
Schematic illustrating cherries and the calculation of Sackin's statistic for a symmetric (*a*) and an asymmetric (*b*) six-taxon cladogram.

This model assumes that the rate of coalescence between any two lineages is the same for all pairs of lineages, but varies over time. If the rate of coalescences varies between lineages at a given time, then this may have an impact on the shape of the tree [17]. Hence, deviations of the shape of an inferred tree from that expected under the coalescent model suggests that additional biological complexity may need to be considered. There are a number of different measures of tree shape that can be used for this purpose [18,19], but we focus on two specific measures: the number of ‘cherries’ [15] and Sackin's index [14]. Figure 1 illustrates how these statistics are calculated for two small trees, one symmetric and one asymmetric. Measures of tree shape tend to consider another stochastic process that generates trees, a linear birth or the Yule process [20]; however, as this model gives the same probability distribution on cladograms (i.e. the topology of the tree) [21], results on asymmetry for the Yule process also hold for the coalescent model.

Cherries are defined as the number of tips that share a direct ancestor, which are generated when two tips coalesce. The expected number of cherries in a tree with *n* taxa under a Yule or coalescent model is *n*/3 [15]. In an asymmetric tree, tips tend to coalesce with branches deeper in the tree, and there are fewer cherries than expected. We denote the number of cherries as *C*, which is not to be confused with another measure of asymmetry, Colless' index [22].

Sackin's index is a measure of the topological distance from the tips of the tree to the root and is defined as follows. If the distance *d_j_* of a leaf *j* is the number of internal nodes that need to be traversed when following the path from the root of the tree to a leaf *j*, then Sackin's index is the sum of all such paths, 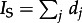. The expectation of Sackin's index for *n* taxa, 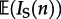, under a Yule or coalescent process [23] is as follows:2.2
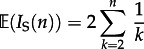
2.3
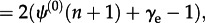
where 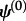 is the polygamma function of order 0, and 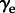 the Euler–Mascheroni constant (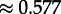). For large *n*, 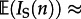 2*n* log(*n*). As Sackin's index increases with sample size, it is often standardized by dividing by the number of sequences. Although this has a direct biological interpretation—the mean root-to-tip distance (in terms of nodes)—we employ a different standardization used by Leventhal *et al.* [13], which is as follows:2.4
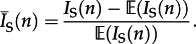


Under the null Yule or coalescent model, 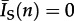, which allows one to assess deviations from the null model more easily. We calculated these tests for two HIV phylogenies, one from an early clinical trial, ACTG 241 [9,24], and another of group M viral sequences sampled from HIV-infected individuals from the Democratic Republic of Congo [25–27]. Both trees show moderate, but statistically significant, evidence of asymmetry (see the electronic supplementary material, figure S1). For both of these datasets, the sequences were sampled at approximately the same time. Although many viral datasets are collected in serial samples, and this can result in more asymmetric trees than if sequences are sampled at a single timepoint (see the electronic supplementary material, figure S2), in order to keep the exposition simple, we will consider sampling at a single timepoint, although the approach taken here can also be extended to serial samples.

The number of cherries and Sackin's index complement each other well, as the number of cherries captures asymmetry in the recent evolutionary past, while Sackin's index captures asymmetry over the entire evolutionary history of the sample, and simulations demonstrate that these statistics are only weakly correlated under the coalescent model (see the electronic supplementary material, figure S3).

## 3. Tree shape in a simple model of HIV infection

**Figure 2.**
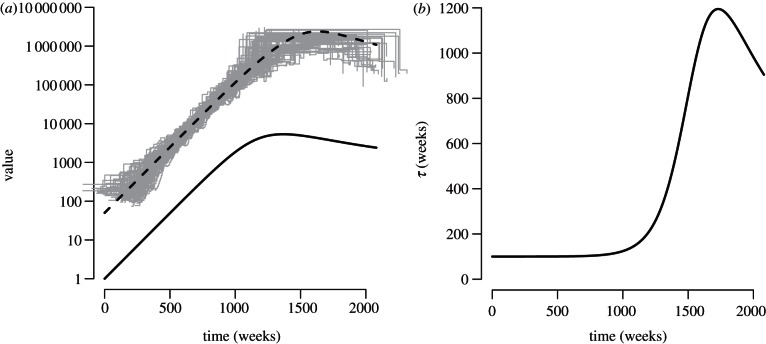
(*a*) Dynamics of the number of infected individuals, *I* (black line), and *ν* = *Iτ*/2 (dashed line) over time in weeks based on equations (3.1)–(3.2), as well as estimates of ‘scaled effective population size’ obtained from applying a Bayesian skyride (grey) to simulated data generated from a forwards-time stochastic version of the model, with 100 replicates. (*b*) Dynamics of the mean generation time, *τ*. Parameter values and initial conditions are as follows: *β* = 0.01, *c* = 1, 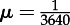, 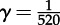, 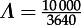, *S*(0) = 9999, *I*(0) = 1, with a simulation time of 40 years. Simulations of the differential equations were performed using the simecol library [29] in R [30], fitting of the skyline plot used the INLA library [31], while the stochastic simulations were performed using SimPy v. 1.9.1 in Python (see [3] for more details). Simulations were conditioned on reaching a quasi-equilibrium state, and registered by aligning the peaks of the simulated number of infected individuals to the peak of infected individuals from the ordinary differential equations. Code to perform simulations is available from http://code.google.com/p/simonfrost.

To investigate how the shape of a viral phylogeny is linked to transmission, we first considered a simple model commonly used to study the spread of HIV among men who have sex with men (for a comparison of the deterministic and stochastic version of this model, see Jacquez & Simon [28]). If *S* denotes the number of susceptible individuals and *I* denotes the number of infected individuals, the rates of change of *S* and *I* are as follows:3.1
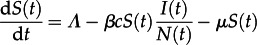
and3.2
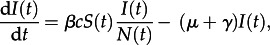
where3.3
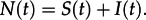
Here, *β* is the per-contact probability of infection, *c* the contact rate, *μ* represents the natural mortality rate, *γ* denotes the excess mortality caused by infection, and **Λ** is the rate of immigration/birth of new susceptibles. The dynamical behaviour of the model depends on the value of the basic reproductive number 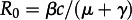. If *R*_0_ > 1 in this model, the number of infected individuals initially increases exponentially, plateaus, and finally reaches an equilibrium (figure 2*a*).

### (a) The number of lineages as a function of time

For the model (3.1)–(3.2), the phylogenetic structure can be captured by the number of lineages as a function of time (NLFT), denoted *A*(*s*), where *S* is time going backwards from the present to the past. A differential equation describing the dynamics of *A* can be derived by first recognizing that the NLFT decreases as a consequence of transmission, but only if both lineages involved in the transmission are sampled.

Let *𝒜*(*s*) denote the set of lineages that are ancestral to the sample at time *s*, so that 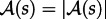. *𝒰*(*s*) will denote the set of lineages which are *not* ancestral to the sample and will have cardinality *U*(*s*). Lower-case symbols will denote elements of these sets: *a* ∈ *𝒜* and *u* ∈ *𝒰*. 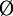 will denote the removal of a lineage. At each internal node of the tree, we denote the types of daughter lineages *i* and *j* and the state of the parent *k* using the notation 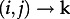. The possible types of transition as we go backwards in time are as follows:

**Table:** 

transition	*ΔA*	*ΔU*	rate
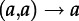	−1	0	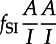
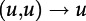	0	−1	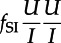
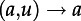	0	−1	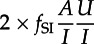
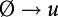	0	+1	*f_IØ_I*

The above transitions assume that the population sizes for *I*, *A* and *U* are large, such that 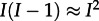, etc., and we consider sampling with replacement for the coalescence of lineages. The first type of transition, 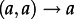 reflects the decrease in lineages when there is an infection involving two sampled lineages. Infections where neither the source nor the recipient individual are sampled do not affect the number of lineages in the sample, and so we do not need to consider transitions of the form 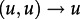 for the NLFT. The transition 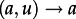 occurs either when a sampled individual infects another individual but we do not sample the latter individual, or when an unsampled individual infects a sampled individual. These transitions reflect what we have described as an ‘invisible’ transmission [32,33]. While such transitions do not affect the number of lineages, for structured populations they may result in a change in state of the lineage, and so are important for more complex models, which we will demonstrate later. The removal of lineages, for example by death or recovery of infected individuals, while changing the number of unsampled lineages (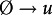), does not directly affect the number of sampled lineages, as the transitions 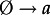 is not possible; in addition, the transition 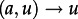 is not possible. These transitions suggest the following set of differential equations for the dynamics of *A* and *U*:3.4
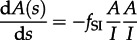
and3.5
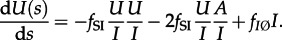


Transitions in this model are more complex than those considered in a simple Wright–Fisher model. Firstly, an infection gives rise to another through transmission, such that there are overlapping ‘generations’. Secondly, sampling effects enter into the transition rates. Nevertheless, there is a one-to-one correspondence of the coalescence rate in this epidemiological model with that in standard population genetics models widely used in viral phylodynamic studies. In a haploid Wright–Fisher model, the dynamics of the effective population size *N*_e_ is in units of generations. The resulting estimates from models fitted to phylogenies where branch lengths are in real time are usually interpreted as *N*_e_*τ*, or the ‘scaled effective population size’, where *N*_e_ is the ‘effective number of infections’ and *τ* the generation time. For the model given by equations (3.1)–(3.2), the coalescence rate is the same as a haploid Wright–Fisher model if we define the number of infected individuals *I*/2 as the ‘effective number of infections’ and the generation time 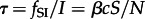 (the incidence-to-prevalence ratio [34]), with the ‘scaled effective population size’ being *Iτ*/2. The use of the term ‘generation time’ here, in a population genetics context, should not be confused with epidemiological interpretations of the generation time [35,36], which is defined at the individual level, as the time between the infection time of an infected person, and the infection time of his or her infector, rather than the average time between infections at a given time at the population level.

Figure 2*a* illustrates the dynamics of *ν* = *Iτ*/2 over time in relation to the number of infected individuals *I* for the model given by equations (3.1)–(3.2). This demonstrates that *ν* is out of phase with *I*, and also exhibits differences in the magnitude of fluctuations. We also fitted a ‘Bayesian skyride’ model [37] to simulated coalescent intervals generated from a forwards-time, stochastic, discrete event version of the model, using a fast approximation that is fitted directly to a phylogenetic tree [38]. Such non-parametric models for *ν* tend to smooth out fluctuations, as well as underestimating *ν* at a given timepoint, as they average *ν* over a time period as the harmonic mean [39]. Nevertheless, the skyride performs well in identifying the overall trajectory of *ν*.

These results argue that the term ‘effective number of infections’ is potentially misleading [3], as it implies that the ancestral size function *ν* is directly proportional to the number of infected individuals. This is not generally the case, owing to a time-varying generation time *τ*(*t*) over the course of an epidemic (figure 2*b*), which is short during the early stages of an epidemic, and becomes longer as the number of susceptible individuals becomes limiting. However, there are time periods, such as the case of exponential growth, and at endemic equilibrium, where the generation time is constant, and hence *ν* is proportional to the number of infected individuals [3,40].

### (b) The number of leaves and cherries

While the distribution of coalescent intervals is sufficient for inference of *ν* under simple models, this is not the case for more realistic models that incorporate heterogeneity. As a prelude to discussing these models, we consider the number of cherries in the homogeneous model. As cherries are generated when two tips coalesce, we consider the dynamics of tips and internal branches separately. *ℒ*(*s*) and *ℬ*(*s*) will denote the set of tips and internal branches, with cardinality *L*(*s*) and *B*(*s*), respectively. As before, lower-case symbols will denote elements of these sets. We denote the cumulative number of cherries as *C*, and consider the following transitions backwards in time:

**Table:** 

transition	*ΔL*	*ΔB*	*ΔC*	rate
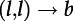	−2	+1	+1	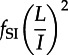
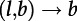	−1	0	0	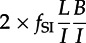
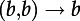	0	−1	0	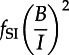

The rationale for this scheme is as follows. When two leaves coalesce, they form a single branch, as well as a cherry. When a leaf and a branch coalesce, this either results in the loss of a leaf, or a loss of a branch and a change of state from a leaf to a branch; both of these occur at the same rate, and result in the same net changes in *L* and *B* (hence the factor of two). When two branches coalesce, this results in the loss of a branch. Consideration of the dynamics of tips also allows us to consider the proportion of lineages that cluster with at least one other sequence, a common approach when analysing HIV phylogenies [5], and is related to the concept of an operational taxonomic unit. The proportion of unclustered tips is 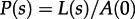, and the distribution of tip lengths is 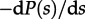. The mean number of taxa per cluster [9], *M*, is included for completeness. If *A*(0) sequences are sampled at a single timepoint *s* = 0, then the initial conditions are *L*(0) = A(0), *B*(0) = 0, *C*(0) = 0 and *M*(0) = 1. This leads to the following set of differential equations for *L*, *B*, *C* and *M*:3.6
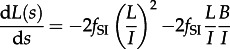
3.7
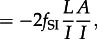
3.8
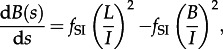
3.9
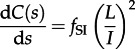
3.10
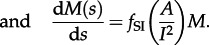


The total number of cherries in a tree is simply the solution of *C*(*s*) at the time to the most recent common ancestor (TMRCA). The only subtlety that arises is the calculation of the TMRCA. In a standard coalescent framework, the TMRCA is the time at which the last two lineages coalesce; in an epidemiological model, this is the time at which the first transmission takes place involving two infected individuals ancestral to the sample, which may occur after the first transmission by the first infected individual in the population. We make the approximation that the TMRCA is the time at which *A* = *L* + *B* = 1.

Theory based on extended Polya urn models [15] has shown that the expected number of cherries in a tree generated by a Yule or coalescent process is *n*/3, where *n* is the number of sequences. We considered the dynamics of cherries for the simple HIV model at endemic equilibrium. If we define a constant 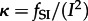, then the solution of equations (3.4)–(3.9) for *A*(*s*), *L*(*s*) and *C*(*s*) is as follows:3.11
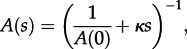
3.12
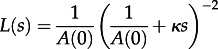
3.13
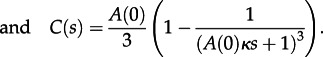


The time to the most recent common ancestor, 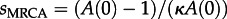, is the solution of *A*(*s*) = 1 for *S*, and the total number of cherries, 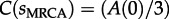 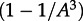; for large *n*, 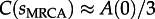, i.e. the total number of cherries in the differential equation model is approximately the same as the mean from a Yule or coalescent process, with only a negligible difference for sample sizes typical of many viral studies, in the order of a hundred or more.

### (c) Sackin′s index

**Figure 3.**
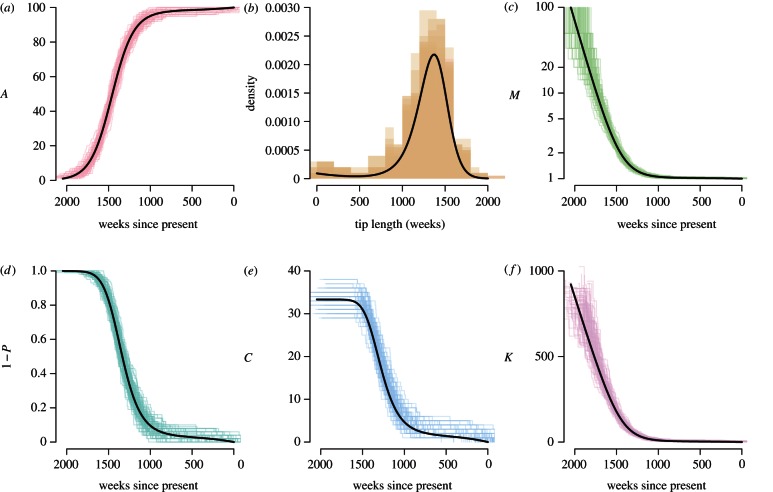
Dynamics of (*a*) the number of lineages, *A*, (*b*) the distribution of tip lengths, (*c*) the mean cluster size, *M*, (*d*) the fraction of sequences clustered, 1−*P*, (*e*) the number of cherries, *C*, and (*f*) Sackin′s index, *K*, for the simple model of HIV infection given by equations (3.1)–(3.2). Parameter values, initial conditions, and simulations are as in figure 2.

As Sackin′s index is the number of internal nodes (including the root) from each tip, summed over all tips, to obtain an approximation for Sackin′s index we need to consider the coalescence rate, 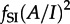, and the expected change in Sackin′s index given a coalescence, which is 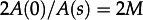, where *M* is the mean cluster size; the factor of two arises due to coalescent events affecting the counts for two lineages. A differential equation for *K*(*s*), the cumulative value of Sackin′s index at time *S* in the past (where *K*(0) = 0), is as follows:3.14
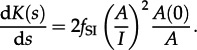



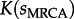
 provides an approximation for Sackin′s statistic. Considering the simple HIV model at equilibrium, substituting 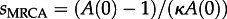 into equation (3.14) gives 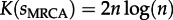. Although as the number of sequences tends to infinity, 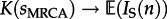, the difference between 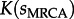 and 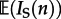 (on the order of 5% for sample sizes in the hundreds) is large enough that we standardize 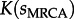 by 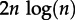 rather than 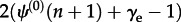, i.e. 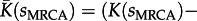 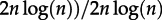. Figure 3 demonstrates the dynamics of these statistics for the model given by equations (3.1)–(3.2), which show excellent correspondence with results obtained with forwards-time stochastic simulations.

## 4. Heterogeneity and tree shape

The model given by equations (3.1)–(3.2) considers only a single type of susceptible and a single type of infected individual. More generally, we can consider models that include heterogeneity between individuals. Examples of such heterogeneity include differences in infectivity at different times since infection, differences between hosts in contact rates, and geographical heterogeneity. Such heterogeneity can have a profound effect on the transmission dynamics. Incorporating heterogeneity in our phylodynamic models presents additional challenges, as we need to consider ancestral lineages for each type of infected individual, and coalescences between lineages of both the same and different types.

### (a) The number of lineages as a function of time

We begin by considering the dynamics of the total number of lineages of each type for a two-type system, although these results can easily be extended to more than two types. Considering forwards time, we define a time-varying matrix *F*(*t*), comprising elements *f_ij_*(*t*), the rate at which a lineage of type *i* generates another of type *j*, and a matrix *G*(*t*), comprising elements *g_ij_*(*t*), the rate at which a lineage of type *i* changes to one of type *j*. These matrices are used to express the transition rates for changes in the number of ancestral lineages of different types [32], which for a two-type system are as follows:

**Table:** 

transition	*ΔA*_1_	*ΔA*_2_	rate
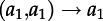	−1	0	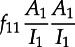
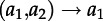	0	−1	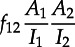
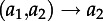	−1	0	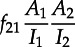
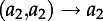	0	−1	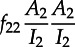
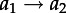	−1	+1	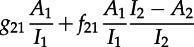
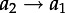	+1	−1	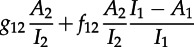

This leads to the following differential equation for the dynamics of *A*_1_(*s*), with an analogous equation for 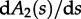:
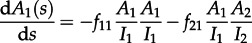

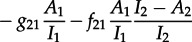
4.1
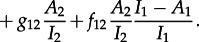


### (b) The number of tips and cherries

Derivation of the dynamics of tips in this two-type system requires us to consider four types of tips, *l*_11_, *l*_21_, *l*_12_ and *l*_22_, based on the (unobserved) state at time *S* in the past (referred to by the first subscript) and the (observed) initial state at the time of sampling. As for simplicity, we consider sampling at a single timepoint, this is at *s* = 0. For example, transitions for the dynamics of tips involving *l*_11_ are as follows:

**Table:** 

transition	*ΔL*_11_	*ΔL*_21_	*ΔL*_12_	*ΔL*_22_	rate
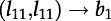	−2	0	0	0	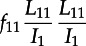
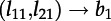	−1	−1	0	0	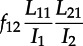
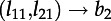	−1	−1	0	0	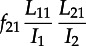
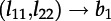	−1	0	0	−1	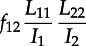
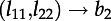	−1	0	0	−1	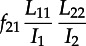
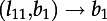	−1	0	0	0	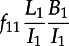
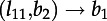	−1	0	0	0	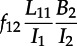
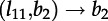	−1	0	0	0	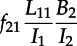
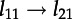	−1	+1	0	0	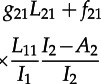
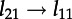	+1	−1	0	0	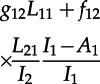

Consideration of these transitions leads to the following differential equation for the dynamics of tips *L*_11_, with analogous expressions for the dynamics of the other tips:
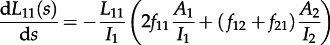

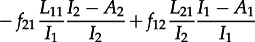
4.2
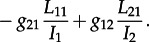


The first line of equation (4.2) represents coalescence of tips, the second ‘invisible’ transmissions, which result in a change in state and the third migration events. The fraction of unclustered lineages that are in state *j* at the tips of the tree, and are in state *i* at some time *s* in the past, *P*_*ij*_(*s*), can be obtained from the above in a similar fashion as in the single-population model.

In this two-type system, there are three types of cherries, which we denote by *c_ij_*. The rates of coalescence of different types of tip (*l*_11_, *l*_12_, *l*_21_ and *l*_22_), and the types of cherry generated are as follows:

**Table:** 

transition	rate	cherry
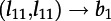	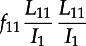	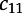
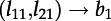	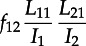	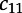
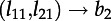	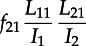	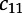
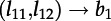	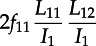	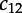
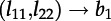	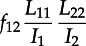	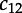
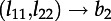	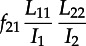	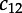
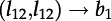	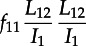	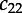
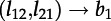	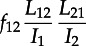	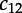
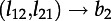	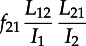	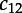
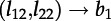	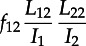	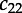
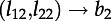	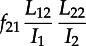	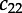
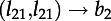	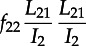	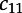
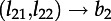	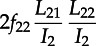	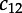
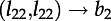	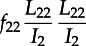	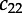

It is important to note that we have to consider both lineages as potential ‘sources’ of infection when considering coalescence between tips of different types, hence the factor of two for 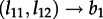 and 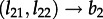. Consideration of these transitions gives rise to the following differential equations for the dynamics of the number of cherries:4.3
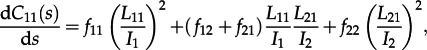
4.4
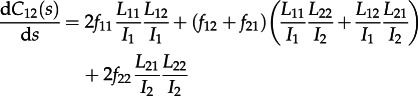
and4.5
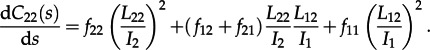


The total number of cherries, 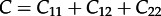 gives a measure of tree asymmetry, which can be compared against the null of 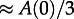. To facilitate comparison of trees with different numbers of tips, *L*(0) = *A*(0), we define a normalized number of cherries, 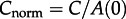.

#### (i) The composition of cherries as a measure of clustering

Capturing how different types cluster together on a tree, i.e. co-clustering, is difficult, as—except at the tips of the tree—the type of a lineage is not directly observable. Previously, we have derived equations for the correlations in numbers of sequences of different types in a cluster [33]. Here, we consider clustering in terms of the composition of different types of cherries, with relatively low values of *C*_12_ being indicative of separation between types. We define the following measure of assortativity, based on that of Newman [41]. We denote a matrix *E* with elements *e_ij_* as follows:4.6
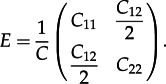


The *assortativity coefficient*, *r*, is defined as follows, where 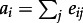 and 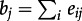:4.7
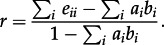
Under a null panmictic model, *r* = 0, while for a model where types are completely separated, *r* = 1. An estimate of *r* can also be obtained directly from a viral phylogeny.

### (c) Sackin′s index

Extending our approximation for Sackin′s index (equation (3.14)) to two subpopulations is relatively straightforward, except now we have to consider three different types of coalescence. When lines of type *i* and *j* coalesce, they produce a clade with a mean number of descendants 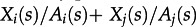, where *X_i_*(*s*) denotes the number of taxa descended from all extant lineages of type *i* at time *s* in the past, with 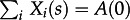. Such clades are produced at the rate 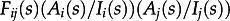. This leads to the following differential equation for the cumulative Sackin index until time *s*:
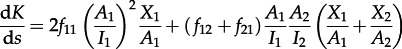
4.8
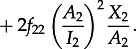


In order to aid comparison with the simple model without heterogeneity, we derive a normalized version of *k*, 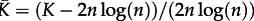.

The dynamics of *X*_1_(*s*) can be described with the following equation, with an analogous equation for *X*_2_(*s*), with initial conditions 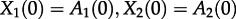:
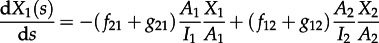
4.9
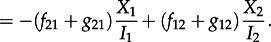
This equation simply captures flow between the states, either by coalescent events (captured by the matrix *F*) or by ‘migration’ between states (captured by the matrix *G*). To verify the deterministic approximations, and to determine the variability in tree shape due to finite sample size, we also simulated trees using an approximation to the coalescent in structured populations developed by Volz [32], which takes the matrices *F* and *G* and the numbers of infected individuals, *I*_1_ and *I*_2_, at different time points as input.

## 5. Applications

To determine how structure and sampling affects phylodynamic patterns, in terms of the number of lineages over time, the extent to which sequences cluster and co-cluster, and the extent of tree asymmetry, we now consider two specific models of HIV that incorporate heterogeneity, either in infectiousness over the course of infection or differences between groups in contact rates.

### (a) Acute and chronic HIV infection

The infectiousness of HIV-1 is thought to be much higher during acute infection than during chronic infection [42]. Previously, we have analysed models of HIV transmission that include acute and chronic infection [9,32,33]. We recap some of the main results here, as well as extending them to consider more tree statistics. We denote the number of acutely infected individuals by *I*_1_, and the number of chronically infected individuals by *I*_2_. We allow acutely infected individuals to have a different per-act probability of infecting a susceptible person, *β*_1_, which we assume to be higher than the probability for a chronically infected person, i.e. 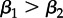. We assume that acute infection progresses to chronic infection at rate *α*, and that acutely infected individuals do not suffer any excess mortality due to HIV infection. These generalizations to the simple HIV model result in the following set of differential equations:5.1
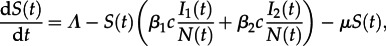
5.2
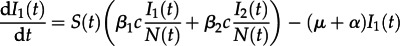
5.3
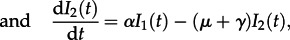
where5.4
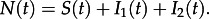


The matrices *F* and *G* for this model are as follows:5.5
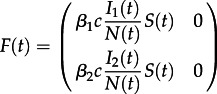
and5.6
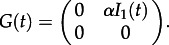


### (b) A model with risk structure

To investigate the effects of heterogeneity in contact rates between individuals, we considered a model with two groups of individuals with different contact rates, *c_i_*, with the fraction of contacts made by a person in group *i* with a person in group *j* denoted by *p_ij_* [43].5.7
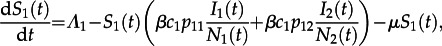
5.8
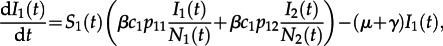
5.9
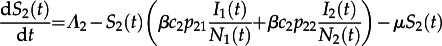

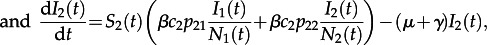
where5.11
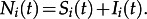


A number of assumptions can be made regarding the fraction of contacts of a person in group *i* with a person in group *j*, *p_ij_*. A common assumption is proportionate mixing, in which the fraction of the contacts of group *i* with group *j* is equal to the fraction of the total contacts made by the population that are due to group *j*, such that 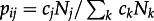. A more general formulation, that allows a wider range of mixing matrices, is the preferred mixing structure described by Jacquez *et al.* [43], in which a fraction *ρ*_*i*_ of the contacts of group *i* are reserved for within-group contacts. The elements *p_ii_* and *p_ij_* (*i* ≠ *j*) under this model are as follows (note that this corrects an error in the term for *p_ij_* reported in Jacquez *et al.* [43]):5.12
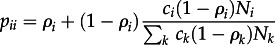
and5.13
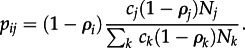


If *ρ*_*i*_ = 0 for all *i*, then the contact matrix simplifies to proportionate mixing. The matrices *F* and *G* for this model are as follows:5.14
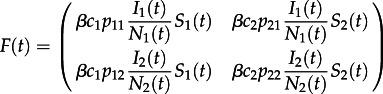
and5.15
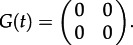


For both the acute/chronic model, and the differential risk model, the differential equations captured the mean number of cherries, the assortativity coefficient and Sackins index calculated from simulations of multiple trees, at a fraction of the computational burden (see the electronic supplementary material, figures S4–S6).

### (c) Tree shape and structure

**Figure 4.**
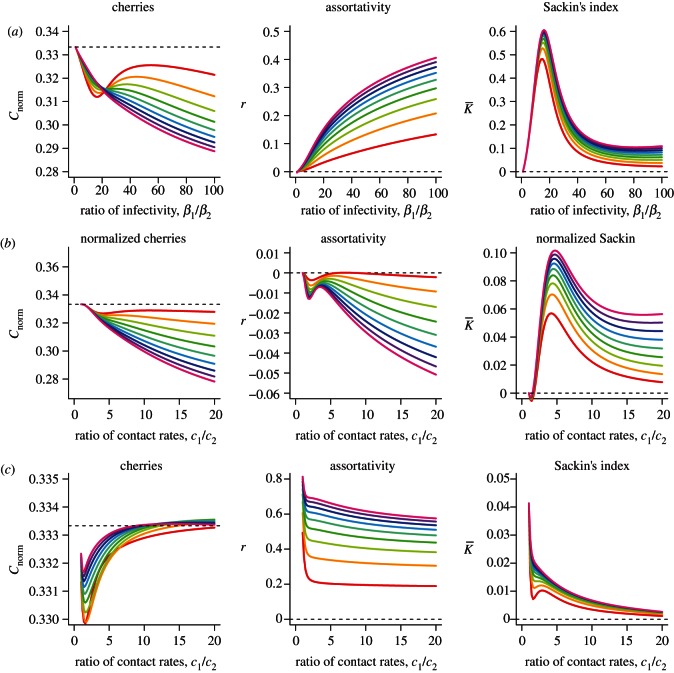
Asymmetry and clustering assuming a range of sampling fractions, from 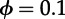 (red) to 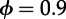 (violet) in steps of 0.1, for different values of the relative infectiousness of acute infection (*a*), and the relative contact rate in the differential risk model, assuming either (*b*) proportionate mixing (*ρ* = 0) or (*c*) preferential mixing (*ρ* = 0.9). Parameter values for the acute/chronic model are as follows: *c* = 1, 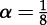, 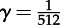, 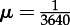, 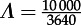, *S*(0) = 9999, *I*_1_(0) = 1, *I*_2_(0) = 0. The infectivity parameters *β*_i_ were constrained such that 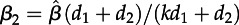 and 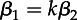, where 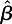 is the mean infectiousness (with 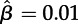), *d_i_* the mean duration of stage *i* and *k* the fold increase in infectiousness during acute infection. Parameter values for the differential risk model are *β* = 0.01, *c*_2_ = 1, 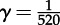, 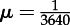, 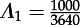, 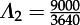, *S*_1_(0) = 999, *I*_1_(0) = 1, *S*_2_(0) = 9000, *I*_2_(0) = 0. The simulation time is 30 years, with weekly timesteps, assuming 52 weeks per year.

We simulated the acute/chronic model and the differential risk model, assuming either proportionate or preferential mixing, for a range of sample fractions, 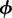, from 0.1 to 0.9. Model outputs for the number of cherries, the assortativity coefficient and Sackin's index at a fixed time of sampling are shown in figure 4.

For the acute/chronic model, we considered a range of values for the relative infectiousness of acute and chronic HIV infection, while maintaining the same mean infectiousness over the infection period. Assortativity increased with higher infectiousness of acute infection, in line with our previous results examining the composition of clusters [33]. Although higher infectiousness resulted in more asymmetric trees, this depended on both the sample fraction and the choice of statistic in a nonlinear way. Sackin's index showed the greatest evidence of asymmetry for intermediate values for the relative infectiousness of acute infection and was generally insensitive to sampling fraction. In contrast, when the sample fraction was high, asymmetry, as measured by a low number of cherries, was the greatest for high infectiousness during acute infection.

For proportionate mixing, the differential risk models over a range of contact rates for the high-risk population relative to the low-risk population demonstrated asymmetry similar in magnitude to those of the acute/chronic model, in terms of the number of cherries, but showed less extreme values for Sackin's index. Assortativity was generally low, with small negative values of the assortativity coefficient for greater relative contact rates for the high-risk group. However, although variation in contact rates could result in asymmetric phylogenies under proportionate mixing, this effect was almost completely eliminated when mixing was preferential (*ρ* = 0.9), as such population subdivision limits the impact that individuals with a high contact rate can have on the entire viral phylogeny. Also in contrast to proportionate mixing, assortativity was much more marked when mixing was preferential. The assortativity coefficient, *r*, was relatively insensitive to contact rate variation, being mainly driven by the mixing between the high- and low-risk groups, captured by the parameter *ρ* (results not shown), although the assortativity of different types of infected individuals, *r*, may be much less than the assortativity of different types of all individuals, *ρ*, especially for low sample fractions.

## 6. Discussion

We have extended our previous differential equation-based framework for modelling the NLFT to consider tree asymmetry and, in the case of structured population models, co-clustering of different states. Of note is that our models generate trajectories of measures of asymmetry and assortativity over evolutionary time, rather than just summary measures over the whole tree. We have also presented examples of how heterogeneity in the susceptible and/or infected individuals can result in different phylodynamic patterns. The two models presented here have a wide range of applications. For example, the model used for acute and chronic HIV infection can also be used to consider a simple form of treatment, where *I*_1_ and *I*_2_ represent the number of untreated and treated individuals, respectively, and *α* represents the rate of going on treatment, while the model of different risk groups can be used to examine heterosexual spread of HIV, by setting *c_ii_*, *i* = 1,2 to zero, or a spatial model, where ‘migration’ of infections occurs via transmission between individuals in different geographical areas.

Given the nonlinearities in the system, it is hard to develop scenarios where the impact of a single parameter can be examined. For example, changing the relative infectiousness of acute infection changes the whole trajectory of the epidemic, such that sampling at a fixed time since the introduction is not strictly comparable across parameter values. As our models comprise a relatively small number of differential equations, which can be simulated quickly, they are well suited for exploring how tree shape is affected by population structure. In contrast, simulating trees may be extremely time consuming, especially for large numbers of taxa, and large numbers of simulations for a given parameter set may be needed, owing to the high variability in many tree shape statistics.

Our results suggest that in many cases, the level of asymmetry of the tree may be rather insensitive to the underlying population structure. This is not particularly surprising for a number of reasons, including the relatively weak selection of HIV-1 at the population level [2], the averaging of asymmetry over the entire tree, and that the risk among those infected is likely to be higher and less variable among infected individuals than susceptible individuals. Given these considerations, it is somewhat surprising that Leventhal *et al.* [13] found asymmetry in their analysis of the Swiss HIV epidemic, especially as the overall phylogeny comprised distinct risk groups, which our results suggest generates *less*, not more asymmetry. This may be due to biases in when each risk group was sampled, and/or the unusually high sampling fraction in this epidemic (30–40%). Indeed, the three largest transmission clusters, which were more homogeneous in terms of risk (one associated with heterosexual risk/injection drug use and two clusters associated with men who have sex with men) showed much lower asymmetry (*I*^−^_S_<0.5). Our models also show that factors other than contact rate, such as high infectiousness during acute infection, may have a more dramatic impact on asymmetry; while high-risk groups may be at a minority in a population, all infected individuals go through a period of increased infectiousness during acute infection. Moreover, as sequences sampled at different times will generate more asymmetric trees for rapidly evolving pathogens such as HIV-1 (see the electronic supplementary material, figure S2), measures of asymmetry may be difficult to interpret for serial samples, which are commonplace in HIV-1 phylogenetic studies, and difficult to compare between studies that have different temporal sampling patterns.

The composition of cherries may be highly informative about patterns of mixing between populations, provided that the sample size is sufficient to include representatives from all groups. However, in order to calculate the composition of cherries, we need to specify subpopulations *a priori*, and this may be difficult to perform, especially for variables such as sexual contact rates. Although, ideally, other data such as behavioural data should be collected in order to identify risk groups, as clustering is also related to contact rates, it may be possible to identify individuals with higher contact rates based on patterns of clustering. However, patterns of clustering have to be interpreted carefully, as differences in clustering may also be driven by differences in the time since infection at which samples are taken ([33]; electronic supplementary material, figure S7) and by the underlying frequencies of the groups (see the electronic supplementary material, figure S8).

Our simulations assumed a random sample of taxa across all groups. In practice, random sampling of infected individuals may not be feasible, or in some cases it may even be desirable to oversample particular groups. For example, while our model of acute and chronic HIV infection predicts increasing assortativity as the assumed relative infectiousness during acute infection increases, it may be difficult to test this empirically, as generally acutely infected individuals are relatively infrequent, and sampling variation in the assortativity coefficient may be high (see the electronic supplementary material, figures S4–S6). Our framework can accommodate over- or under-sampling of specific groups, although prior information on the size of each group is highly desirable in order to make accurate inferences.

We have focused on developing and simulating phylodynamic models, rather than inferring parameter values of these models from sequence data. As highlighted in our discussion of the simple HIV model, some simple epidemiological models are just special cases of the time-varying coalescent model, for which methods of inference are well established. While the theory presented for structured models can also be used as a basis for inference, full likelihood-based fitting may be computationally intensive, and approximations to the likelihood may be required [32]. The models presented here, which can generate a number of summary measures of phylogenetic structure, can be used as the basis for Approximate Bayesian Computation (ABC) approaches [44], in which parameter values are found that generate simulated data that resemble the observed data. The use of more biologically realistic phylodynamic models can be used not only to determine whether a population deviates from random mixing [13], but also to determine the type of population structure. By linking asymmetry, assortativity and the number of lineages through time, bespoke models of viral phylodynamics may be able to provide rich insights into the dynamics of viral transmission.

## References

[1] Pybus OG Rambaut A 2009 Evolutionary analysis of the dynamics of viral infectious disease. Nat. Rev. Genet. 10, 540–550 (doi:10.1038/nrg2583) 19564871 10.1038/nrg2583 PMC7097015

[2] Grenfell BT 2004 Unifying the epidemiological and evolutionary dynamics of pathogens. Science 303, 327–332 (doi:10.1126/science.1090727) 14726583 10.1126/science.1090727

[3] Frost SDW Volz EM 2010 Viral phylodynamics and the search for an ‘effective number of infections’. Phil. Trans. R. Soc. B 365, 1879–1890 (doi:10.1098/rstb.2010.0060) 20478883 10.1098/rstb.2010.0060 PMC2880113

[4] Hué S Pillay D Clewley JP Pybus OG 2005 Genetic analysis reveals the complex structure of HIV-1 transmission within defined risk groups. Proc. Natl Acad. Sci. USA 102, 4425–4429 (doi:10.1073/pnas.0407534102) 15767575 10.1073/pnas.0407534102 PMC555492

[5] Lewis F Hughes GJ Rambaut A Pozniak A Brown AJL 2008 Episodic sexual transmission of HIV revealed by molecular phylodynamics. PLoS Med. 5, e50 (doi:10.1371/journal.pmed.0050050) 18351795 10.1371/journal.pmed.0050050 PMC2267814

[6] Rambaut A Pybus OG Nelson MI Viboud C Taubenberger JK Holmes EC 2008 The genomic and epidemiological dynamics of human influenza A virus. Nature 453, 615–619 (doi:10.1038/nature06945) 18418375 10.1038/nature06945 PMC2441973

[7] Smith GJD 2009 Origins and evolutionary genomics of the 2009 swine-origin H1N1 influenza A epidemic. Nature 459, 1122–1125 (doi:10.1038/nature08182) 19516283 10.1038/nature08182

[8] Bedford T Cobey S Beerli P Pascual M 2010 Global migration dynamics underlie evolution and persistence of human influenza A (H3N2). PLoS Pathog. 6, e1000918 (doi:10.1371/journal.ppat.1000918) 20523898 10.1371/journal.ppat.1000918 PMC2877742

[9] Volz EM Pond SLK Ward MJ Brown AJL Frost SDW 2009 Phylodynamics of infectious disease epidemics. Genetics 183, 1421–1430 (doi:10.1534/genetics.109.106021) 19797047 10.1534/genetics.109.106021 PMC2787429

[10] Rasmussen DA Ratmann O Koelle K 2011 Inference for nonlinear epidemiological models using genealogies and time series. PLoS Comput. Biol. 7, e1002136. (doi:10.1371/journal.pcbi.1002136). 10.1371/journal.pcbi.1002136 PMC3161897 21901082

[11] Bloomquist EW Lemey P Suchard MA 2010 Three roads diverged? Routes to phylogeographic inference. Trends Ecol. Evol. 25, 626–632 (doi:10.1016/j.tree.2010.08.010) 20863591 10.1016/j.tree.2010.08.010 PMC2956787

[12] Zárate S Pond SLK Shapshak P Frost SDW 2007 Comparative study of methods for detecting sequence compartmentalization in human immunodeficiency virus type 1. J. Virol. 81, 6643–6651 (doi:10.1128/JVI.02268-06) 17428864 10.1128/JVI.02268-06 PMC1900087

[13] Leventhal GE. 2012 Inferring epidemic contact structure from phylogenetic trees. PLoS Comput. Biol. 8, e1002413. (doi:10.1371/journal.pcbi.1002413) 10.1371/journal.pcbi.1002413 PMC3297558 22412361

[14] Sackin M 1972 ‘Good’ and ‘bad’ phenograms. Syst. Biol. 21, 225–226 (doi:10.1093/sysbio/21.2.225)

[15] McKenzie A Steel M 2000 Distributions of cherries for two models of trees. Math. Biosci. 164, 81–92 (doi:10.1016/S0025-5564(99)00060-7) 10704639 10.1016/s0025-5564(99)00060-7

[16] Griffiths RC Tavaré S 1994 Sampling theory for neutral alleles in a varying environment. Phil. Trans. R. Soc. Lond. B 344, 403–410 (doi:10.1098/rstb.1994.0079) 7800710 10.1098/rstb.1994.0079

[17] Heard SB Mooers AO 1996 Imperfect information and the balance of cladograms and phenograms. Syst. Biol. 45, 115–118 See http://www.jstor.org/stable/2413517

[18] Mooers AO Heard SB 1997 Inferring evolutionary process from phylogenetic tree shape. Q. Rev. Biol. 72, 31–54 See http://www.jstor.org/stable/3036810

[19] Agapow PM Purvis A 2002 Power of eight tree shape statistics to detect nonrandom diversification: a comparison by simulation of two models of cladogenesis. Syst. Biol. 51, 866–872 (doi:10.1080/10635150290102564) 12554452 10.1080/10635150290102564

[20] Yule GU 1925 A mathematical theory of evolution, based on the conclusions of Dr J. C. Willis, F.R.S. Phil. Trans. R. Soc. Lond. B 213, 21–87 (doi:10.1098/rstb.1925.0002)

[21] Aldous DJ 2001 Stochastic models and descriptive statistics for phylogenetic trees, from Yule to today. Stat. Sci. 16, 23–34 See http://www.jstor.org/stable/2676778

[22] Colless D 1982 Phylogenetics: the theory and practice of phylogenetic systematics. Syst. Zool. 31, 100–104 (doi:10.2307/2413420)

[23] Kirkpatrick M Slatkin M 1993 Searching for evolutionary patterns in the shape of a phylogenetic tree. Evolution 47, 1171–1181 See http://www.jstor.org/stable/2409983 10.1111/j.1558-5646.1993.tb02144.x 28564277

[24] D'Aquila RT 1996 Nevirapine, zidovudine, and didanosine compared with zidovudine and didanosine in patients with HIV-1 infection. A randomized, double-blind, placebo-controlled trial. Ann. Intern. Med. 124, 1019–1030 8633815 10.7326/0003-4819-124-12-199606150-00001

[25] Vidal N 2000 Unprecedented degree of human immunodeficiency virus type 1 (HIV-1) group M genetic diversity in the Democratic Republic of Congo suggests that the HIV-1 pandemic originated in Central Africa. J. Virol. 74, 10 498–10 507 (doi:10.1128/JVI.74.22.10498-10507.2000) 10.1128/jvi.74.22.10498-10507.2000 PMC110924 11044094

[26] Yusim K 2001 Using human immunodeficiency virus type 1 sequences to infer historical features of the acquired immune deficiency syndrome epidemic and human immunodeficiency virus evolution. Phil. Trans. R. Soc. Lond. B 356, 855–866 (doi:10.1098/rstb.2001.0859) 11405933 10.1098/rstb.2001.0859 PMC1088479

[27] Strimmer K Pybus OG 2001 Exploring the demographic history of DNA sequences using the generalized skyline plot. Mol. Biol. Evol. 18, 2298–2305 (doi:10.1093/oxfordjournals.molbev.a003776) 11719579 10.1093/oxfordjournals.molbev.a003776

[28] Jacquez JA Simon CP 1993 The stochastic SI model with recruitment and deaths. I. Comparison with the closed SIS model. Math. Biosci. 117, 77–125 (doi:10.1016/0025-5564(93)90018-6) 8400585 10.1016/0025-5564(93)90018-6

[29] Petzoldt T Rinke K 2007 simecol: an object-oriented framework for ecological modeling in R. J. Stat. Softw. 22, 1–31 See http://www.jstatsoft.org/v22/i09

[30] R Core Team 2012 R: a language and environment for statistical computing. Vienna, Austria See http://www.R-project.org/

[31] Rue H Martino S Lindgren F 2009 INLA: Functions which allow to perform a full Bayesian analysis of structured (geo-)additive models using Integrated Nested Laplace Approximation, R package v. 0.0.

[32] Volz EM 2012 Complex population dynamics and the coalescent under neutrality. Genetics 190, 187–201 (doi:10.1534/genetics.111.134627) 22042576 10.1534/genetics.111.134627 PMC3249372

[33] Volz EM Koopman JS Ward MJ Brown AL Frost SDW 2012 Simple epidemiological dynamics explain phylogenetic clustering of HIV from patients with recent infection. PLoS Comput. Biol. 8, e1002552 (doi:10.1371/journal.pcbi.1002552) 22761556 10.1371/journal.pcbi.1002552 PMC3386305

[34] White PJ Ward H Garnett GP 2006 Is HIV out of control in the UK? An example of analysing patterns of HIV spreading using incidence-to-prevalence ratios. AIDS 20, 1898–1901 (doi:10.1097/01.aids.0000244213.23574.fa) 16954735 10.1097/01.aids.0000244213.23574.fa

[35] Svensson A 2007 A note on generation times in epidemic models. Math. Biosci. 208, 300–311 (doi:10.1016/j.mbs.2006.10.010) 17174352 10.1016/j.mbs.2006.10.010

[36] Kenah E Lipsitch M Robins JM 2008 Generation interval contraction and epidemic data analysis. Math. Biosci. 213, 71–79 (doi:10.1016/j.mbs.2008.02.007) 18394654 10.1016/j.mbs.2008.02.007 PMC2365921

[37] Minin VN Bloomquist EW Suchard MA 2008 Smooth skyride through a rough skyline: Bayesian coalescent-based inference of population dynamics. Mol. Biol. Evol. 25, 1459–1471 (doi:10.1093/molbev/msn090) 18408232 10.1093/molbev/msn090 PMC3302198

[38] Palacios JA Minin VN 2012 Integrated nested Laplace approximation for Bayesian nonparametric phylodynamics. In Proc. 28th Conf. on Uncertainty in Artificial Intelligence (eds de Freitas N Murphy K), pp. 726–735 Corvallis, OR: AUAI Press

[39] Pybus OG Rambaut A Harvey PH 2000 An integrated framework for the inference of viral population history from reconstructed genealogies. Genetics 155, 1429–1437 10880500 10.1093/genetics/155.3.1429 PMC1461136

[40] Koelle K Rasmussen DA 2012 Rates of coalescence for common epidemiological models at equilibrium. J. R. Soc. Interface 9, 997–1007 (doi:10.1098/rsif.2011.0495) 21920961 10.1098/rsif.2011.0495 PMC3306638

[41] Newman MEJ 2003 Mixing patterns in networks. Phys. Rev. E 67, 026126 (doi:10.1103/PhysRevE.67.026126) 10.1103/PhysRevE.67.026126 12636767

[42] Pilcher CD 2004 Brief but efficient: acute HIV infection and the sexual transmission of HIV. J. Infect. Dis. 189, 1785–1792 (doi:10.1086/386333) 15122514 10.1086/386333

[43] Jacquez JA Simon CP Koopman J Sattenspiel L Perry T 1988 Modeling and analyzing HIV transmission: the effect of contact patterns. Math. Biosci. 92, 119–199 (doi:10.1016/0025-5564(88)90031-4)

[44] Lopes JS Beaumont MA 2010 ABC: a useful Bayesian tool for the analysis of population data. Infect. Genet. Evol. 10, 826–833 (doi:10.1016/j.meegid.2009.10.010) 19879976 10.1016/j.meegid.2009.10.010

